# Abnormal T2-STIR Magnetic Resonance in Hypertrophic Cardiomyopathy: A Marker of Advanced Disease and Electrical Myocardial Instability

**DOI:** 10.1371/journal.pone.0111366

**Published:** 2014-10-30

**Authors:** Giancarlo Todiere, Lorena Pisciella, Andrea Barison, Annamaria Del Franco, Elisabetta Zachara, Paolo Piaggi, Federica Re, Alessandro Pingitore, Michele Emdin, Massimo Lombardi, Giovanni Donato Aquaro

**Affiliations:** 1 Fondazione G. Monasterio Regione Toscana-National Research Council, Pisa, Italy; 2 Cardiologia 2 Azienda Ospedaliera San Camillo-Forlanini, Rome, Italy; 3 Endocrinology Unit, University Hospital, Pisa, Italy; 4 Institute of Clinical Physiology, National Research Council, Pisa, Italy; 5 Multimodality Imaging Section, San Donato, Milano, Italy; University of Minnesota, United States of America

## Abstract

**Background:**

Myocardial hyperintensity on T2-weighted short-tau inversion recovery (STIR) (HyT2) cardiac magnetic resonance (CMR) images has been demonstrated in patients with hypertrophic cardiomyopathy (HCM) and is considered a sign of acute damage. The aim of the current study was to evaluate the relationship between HyT2 and both a) markers of ventricular electrical instability and b) clinical and CMR parameters.

**Methods:**

Sixty-five patients underwent a thorough clinical examination, consisting of 24-h ECG recording and CMR examination including functional evaluation, T2-STIR images and late gadolinium enhancement (LGE).

**Results:**

HyT2 was detected in 27 patients (42%), and subjects with HyT2 showed a greater left ventricle (LV) mass index (p<0.001), lower LV ejection fraction (p = 0.05) and greater extent of LGE (p<0.001) compared to those without HyT2. Twenty-two subjects (34%) presented non-sustained ventricular tachycardia (NSVT) on the 24-h ECG recording, 21 (95%) of whom exhibited HyT2. Based on the logistic regression analysis, HyT2 (odds ratio [OR]: 165, 95% CI 11–2455, p<0.001) and LGE extent (1.1, 1.0–1.3, p<0.001) served as independent predictors of NSVT, while the presence of LGE was not associated with NSVT occurrence (p = 0.49). The presence of HyT2 was associated with lower heart rate variability (p = 0.006) and a higher number of arrhythmic risk factors (p<0.001).

**Conclusions:**

In HCM patients, HyT2 upon CMR examination is associated with more advanced disease and increased arrhythmic burden.

## Introduction

The prevention of sudden cardiac death is the most relevant challenge in patients with hypertrophic cardiomyopathy (HCM) [1]–[4]. The presence of myocardial fibrosis, as evaluated by cardiac magnetic resonance (CMR) imaging with the late gadolinium enhancement (LGE) technique, is associated with the occurrence of non-sustained ventricular tachycardia (NSVT), as observed via 24-h Holter electrocardiography (ECG) recording [5]–[7], and a worse clinical outcome [8]–[10]. However, the vast majority of HCM patients (60–85% prevalence at the first CMR evaluation, increasing up to >95% during follow-up) show LGE [11], which may be considered a nonspecific marker of this disease.

Myocardial hyperintensity upon CMR T2-weighted short-tau inversion recovery (STIR) imaging (HyT2) is a sign of edema that is secondary to acute ischemic or inflammatory damage [12]–[13] and is present in a subset of patients with HCM, where it is likely caused by myocardial ischemia [14]. Myocardial ischemia seems to be associated with microvascular impairment in HCM, where it is considered a trigger for arrhythmic events and has been associated with worse prognoses [15]–[16]. Although the relationship between HyT2 and NSVT was initially reported in patients with HCM [17], [18], it has never been prospectively evaluated. Therefore, the aims of the current study were as follows: a) to assess the relationship between HyT2 and signs of ventricular electrical instability (premature ventricular contractions, PVC, and NSVT), autonomic impairment according to heart rate variability on 24 h-Holter ECG recordings, and the arrhythmic risk score [16] and b) to compare HyT2 to other CMR parameters, such as the presence and extent of LGE, left ventricular (LV) mass index, and maximal LV end-diastolic wall thickness.

### Patients and Methods

We enrolled 69 consecutive patients with HCM based on previously reported criteria undergoing a CMR examination. Three patients were excluded for low-quality images, and 1 was excluded for claustrophobia. Thus, the final population consisted of 65 patients (51 males, 49±17 years). The study was approved by the Ethical Committee of Fondazione G.Monasterio-Pisa. All the patients received and signed informed consent.

### Clinical evaluation

The presence of established risk factors for sudden death in patients with HCM were evaluated, including a family history of sudden death, extreme LV wall thickness (>30 mm), unexplained syncope, non-sustained ventricular tachycardia on an ambulatory Holter ECG recording (>3 ventricular beats at a heart rate >120 beats per min), and an abnormal or “flat” systolic arterial pressure during an exercise stress test [19]. A complete clinical evaluation was performed on the day of CMR examination. Based on the clinical examination, each patient was assigned a New York Heart Association (NYHA) class on the basis of the presence and severity of dyspnea. Other symptoms (syncope, chest pain, palpitations) were also recorded. A 12-lead resting ECG was recorded on the same day.

Patients also underwent a 24-h ECG recording around the time of CMR examination. Conventional ECG analysis was performed, including the following heart rate variability measurements in the time-domain analysis: standard deviation (SD) of the RR intervals, SD of the average normal to normal QRS (NN) interval calculated over periods of 5 min (SDANN), the number of interval differences of successive NN intervals >50 msec divided by the total number of NN intervals (pNN50), and the square root of the mean squared differences of successive NN intervals (RMSSD) [20], [21].

### CMR acquisition protocol

CMR was performed using two 1.5 Tesla systems: a Signa Hdx (General Electric Healthcare, Milwaukee, Wisconsin) and a 1.5 Tesla Magnetom Avanto (Siemens, Erlangen, Germany) with cardiac phased array multichannel coils.

Short axis cine images from the mitral plane valve to the LV apex were acquired using a steady-state free precession pulse sequence with the following parameters: 30 phases, slice thickness 8 mm, no gap, views per segment 8, NEX 1, FOV 40 cm, phase FOV 1, matrix 224×224, reconstruction matrix 256×256, 45° flip angle, and a TR/TE equal to 3.5/1.5.

T2-STIR images were acquired using triple inversion recovery T2-weighted pulse sequence in short axis views and 2 long axis views (vertical and horizontal long axis view) using the following parameters: TR = 2 RR, TE ≈ 70 msec, FOV 40 cm, phase FOV 1, matrix 256×256. LGE images were acquired 10 min after the administration of Gd-DTPA (Magnevist, Schering-AG) with a dosage of 0.2 mmol/kg for the short axis views. An inversion recovery T1-weighted gradient- echo (GRE) sequence was used with the following parameters: field of view 40 mm, slice thickness 8 mm, no gap between each slice, repetition time 4.6 msec, echo time 1.3, a flip angle of 20°, matrix 224×224, reconstruction matrix 256×256, and an excitation number of 1. The appropriate inversion time was set to null for normal myocardium (range 250–300 msec).

### Images post-processing

A commercially available research software package (Mass Analysis, Leyden, The Netherlands) was used to quantify the functional parameters using conventional methods [22]. LV mass index was considered severely increased when it was >3 SD over the upper limit of normality [23]. Maximal LV end diastolic wall thickness was measured as previously described [24].

T2-STIR images were evaluated using a qualitative visual assessment performed by three expert independent investigators who were blinded to each other’s results. The presence of signal abnormalities (HyT2) was established when there was agreement between at least two investigators. Agreement among the three investigators was reached when analyzing the images of 95% patients.

The extent of LGE was measured using a previously validated method [25]. Briefly, the endocardial and epicardial contours in each image were manually traced to identify the LV myocardium in each image. To obtain the SD of the signal noise of the images, a region of interest was placed in the background of the image, near the patient's thoracic wall. The mean signal intensity and SD were measured in this region of interest. Myocardial voxels with a signal intensity higher than the average signal intensity of the region of interest plus 6 SD were considered enhanced [26]. The percentage of enhanced voxels in the entire LV myocardium was measured. The extent of LGE was expressed in grams and the percentage of the LV mass.

### Statistical analysis

Categorical variables were compared using Pearson’s chi-squared test with the continuity correction or Fisher's exact test when appropriate. The Kolmogorov-Smirnov test was used to assess the normality of the data. Pearson’s (r) and Spearman’s (ρ) correlation coefficients were employed for Gaussian and skewed variables, respectively. One-way analysis of variance (ANOVA) and Kruskal-Wallis test, when appropriate, were employed to compare quantitative variables between groups. Logistic regression analysis was used to explore the impact of each variable in a model, with NSVT upon 24-h Holter ECG monitoring as the dependent variable and myocardial abnormalities on T2-STIR, LGE extent, LV mass index, maximal end-diastolic wall thickness, and number of arrhythmic risk factors (excluding NSVT) as independent variables.

A p-value <0.05 was considered statistically significant. Data are presented as continuous variables and proportions (percentages). Continuous variables are expressed as the mean ± SD or the median and interquartile range (IQR), as indicated.

## Results

The clinical characteristics of the HCM population are reported in table 1. All patients were in sinus rhythm; however, previous paroxysmal atrial fibrillation was reported in 16 patients (24%). None of the subjects had severe valvular abnormalities.

**Table 1:** Clinical characteristics of the population.

		Whole population	Hy-T2	No-HyT2	p value
**Population characteristics:**	n(%)	65	27(42)	38(58)	
Age (years)	mean±SD	49±17	52±14	46±17	0.59
Male	n(%)	51 (78)	22(81)	29(76)	0.84
LVOT obstruction	n(%)	15(23)	6(22)	9(24)	0.98
History of paroxysmal atrial fibrillation	n(%)	16(25)	8(30)	8(21)	0.59
Reduced ejection fraction (<50%)	n(%)	6(9)	5(19)	1(3)	0.08
Hypertension	n(%)	32(49)	9(33)	23(61)	0.07
Diabetes Mellitus	n(%)	6(9)	0	6	0.08
Hospitalized	n(%)	11(17)	5(19)	6(16)	0.97
Increased HS-Troponine	n(%)	33(51)	20(74)	13(34)	0.39
**Arrhyhtmic Risk Factors:**					
Family history of SCD	n(%)	11(17)	5(19)	6(16)	0.97
VT at 24 h Holter monitoring	n(%)	22(34)	21(78)	1(3)	<0.001
Resuscitated SCD	n(%)	0	0	0	ns
Maximal wall thickness≥30	n(%)	8(12)	7(26)	1(3)	0.02
Unexplained Syncope	n(%)	6(9)	4(15)	2(5)	0.31
Outflow gradient >30 mmHg	n(%)	11(17)	4(15)	7 (18)	0.86
Abnormal pressure response during effort	n(%)	0	0	0	ns
Patients with 0 risk factors	n(%)	36(55)	3(11)	33(87)	<0.001
Patients with 1 risk factors	n(%)	18(28)	14(52)	4(11)	<0.001
Patients with≥2 risk factors	n(%)	11(17)	10(37)	1(3)	<0.001
**Symptoms:**					
Angina	n(%)	13(20)	7(26)	6(16)	0.42
Syncope	n(%)	6(9)	4(15)	2(5)	0.31
Palpitation	n(%)	26(40)	17(63)	9(24)	0.002
Dyspnea	n(%)	34(52)	11(41)	23(61)	0.18
NYHA class II	n(%)	26(40)	14(52)	12(32)	0.17
NYHA class III-IV	n(%)	5(8)	3(11)	2(5)	0.69
**24 h Holter ECG monitoring:**					
PVC	median(IQR)	37 (9–505)	33(14–1253)	41 (8–474)	0.74
NSVT	n(%)	22	21	1	<0.001
SDNN (ms)	mean±SD	130±51	110±38	154±64	<0.01
SDANN (ms)	mean±SD	95±25	86±25	102±21	0.02
pNN50(%)	mean±SD	8±5	9±6	7±5	0.15
RMSSD(ms)	mean±SD	49±24	48±17	50±30	0.75
**Therapy:**					
Beta-blockers	n(%)	35(54)	17(63)	18(47)	0.29
Calcium antagonists	n(%)	5(8)	4(15)	1(3)	0.13
ACE inhibitors	n(%)	17(26)	7(26)	10(26)	0.98
Antiarrhythmic drugs	n(%)	15(23)	8(30)	7(18)	0.52

LVOT, left ventricular outflow tract; SCD, sudden cardiac death; NYHA, New York Heart Association Class; PVC, premature ventricular complexes; NSVT, non sustained ventricula tachycardia; SDNN, standard deviation of of RR intervals;SDANN, standard deviation of the average normal to normal QRS intervals calculated over periods of 5 min; pNN50, the number of interval differences of successive NN intervals >50 ms divided by the total number of NN intervals;RMSSD, the square root of the mean squared differences of successive NN intervals.

Twenty-two subjects (34%) presented with non-sustained ventricular tachycardia on the 24-h ECG recording, 11 (17%) had a family history of sudden death, and 8 (12%) showed a maximal end-diastolic wall thickness ≥30 mm, while none had a significant blood pressure drop during exercise. Thirty-six patients (55%) had no risk factors for sudden death, 18 (28%) presented one risk factor, and 11 (17%) patients had an arrhythmic risk score ≥2.

### CMR findings

A summary of the CMR results is reported in table 2. The LV mass index was increased in 44 patients (68%) and severely increased in 23 patients (35%) (24). LV systolic dysfunction (LV ejection <55%) was found in 6 patients (9%), and LV dilation was present in two patients (3%).

**Table 2:** CMR parameters.

		Global population	Hy-T2	No-HyT2	p value*
**Population:**	n(%)	65	27(42)	38(58)	
**Septal morphology:**					
** - Reverse septal**	n(%)	30(46)	12(44)	18(47)	0.85
** -Sigmoid**	n(%)	20(31)	8(40)	12(31)	0.63
** -Neutral**	n(%)	9(14)	3(11)	6(16)	0.75
** -Apical**	n(%)	6(9)	2(7)	4(10)	0.65
**Maximal Wall thickness (mm)**	mean±SD	21±6	25±7	19±5	0.63
**LV EDVi (ml/m2)**	mean±SD	73±26	76±35	71±16	0.86
**LV ESVi (ml/m2)**	mean±SD	24±19	28±27	20±9	0.11
**LV Mass index (g/m2)**	mean±SD	112±40	133±47	98±28	**<0.001**
**LV ejection fraction (%)**	mean±SD	69±12	66±15	72±8	**<0.05**
**RV EDVi (ml/m2)**	mean±SD	65±17	62±18	67±17	0.27
**RV ESVi (ml/m2)**	mean±SD	19±8	18±9	19±7	0.76
**RV ejection fraction (%)**	mean±SD	70±7	70±9	71±5	0.63
**LGE presence**	n(%)	55(84)	23(84)	32(84)	0.86
**LGE extent (% of LV mass)**	mean±SD	11±11	16±12	7±8	**<0.001**

LV, left ventricle; EDVi, end-diastolic volume index; ESVi, end-systolic volume index, RV, right ventricle; LGE, late gadolinium enhancement.

LGE was present in 55 patients (84%). Patients with LGE had greater end-diastolic wall thickness (p = 0.05), and a linear relationship was found between the extent of LGE and the LV mass index (ρ = 0.27, p = 0.03).

### T2-STIR, clinical variables and CMR parameters

**Figure 1:**
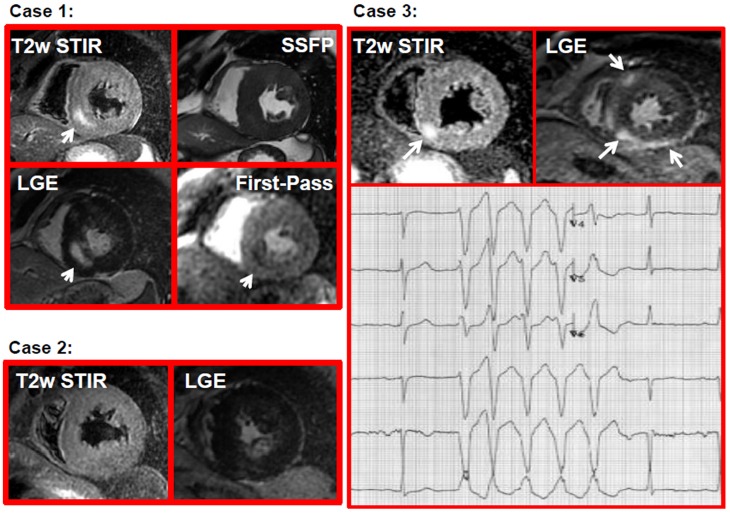
Examples of HCM patients. Case 1: a patient with HCM presenting with HyT2 (arrow in T2-STIR image), myocardial fibrosis (arrow LGE image), and pefusion defect (arrow in the frame of the first pass gadolinium) in the same myocardial segments; Case 2: a patient with HCM having myocardial fibrosis (LGE image) without HyT2 (T2-STIR image); Case 3: a patient with HyT2 (arrow in T2-STIR images) and myocardial fibrosis (arrow in LGE images) having a run of NSVT in the ECG stripe (lower panel).

On T2-STIR images, HyT2 was detected in 27 patients (42%), with no sex (p = 0.62) or age (p = 0.10) differences (Figure 1). The relationships between HyT2 and the clinical and CMR parameters are summarized in tables 1 and 2. As shown in table 2, subjects with HyT2 had a greater LV mass index, lower LVEF and higher extent of LGE than those without HyT2. In addition, the prevalence of HyT2 was higher in patients with severely increased LV mass index (26% vs. 17%, p = 0.004), and all patients presenting with systolic dysfunction had HyT2. The extent of HyT2 was significantly lower than that of LGE (p<0.001). In 24 of 27 patients (88%), HyT2 was found in a myocardial region with LGE, whereas HyT2 and LGE were detected in different myocardial regions in 3 subjects.

### T2-STIR, and electrical instability

During the 24-h ECG, 22 patients (27%) had episodes of NSVT. As shown in table 3, patients with NSVT had higher LGE extents, greater end-diastolic maximal wall thickness, lower LV ejection fraction (64±15% vs. 72±8%, respectively, p = 0.006), greater LV mass index and more arrhythmic risk factors (0.6±0.7 vs. 0.14±0.3, respectively, p = 0.001) than those without NSVT. HyT2 was present in 21 (95%) patients with NSVT (p<0.001); conversely, only 6 of 27 (22%) patients with HyT2 had no NSVT (Figure 1). The presence of LGE was not associated with the occurrence of NSVT (p = 0.35), as 39 patients with LGE (64%) had no NSVT, while all patients with NSVT showed LGE. The SD of RR interval and SDANN upon ECG 24-h ECG monitoring were significantly lower in patients with HyT2 than those without (110±38 msec vs. 154±64 msec, p<0.01, and 102±21 msec vs. 86±25 msec, p = 0.02, respectively). Based on the logistic regression analysis, HyT2 and LGE extent were independent predictors of NSVT upon 24-h Holter ECG recording (table 3).

**Table 3:** Predictors of NSVT.

Indipendent variables				Logistic regression Analysis			
	NSVT	No NSVT	p value	Coefficient	Odds Ratio	95%-C.I.	p-value
**Arrhythmic Risk factors**	0.6±0.7	0.14±0.3	0.001				
**LV mass index**	131±38	103±39	0.007				
**HyT2**	21(95)	1(5)	<0.001	5.1	165	11–2455	<0.001
**Maximal Wall Thickness**	25±6	19±6	<0.001	0.12	1.1	1.0–1.3	<0.001
**Extent of LGE**	18.3±11.9	6.8±7.9	<0.001				

Arrhythmic risk factors: number of arrhythmic risk factors, n ± SD; LV (left ventricular) mass index, expressed in g/m^2^± SD; HyT2, presence of myocardial signal hyperintensity in T2-STIR image, expressed as n(%); Maximal wall thickness in mm ± SD; Extent of LGE, late gadolinium enhancement, in % of LV mass.

## Discussion

Together, our findings led to the following conclusions: 1) HyT2 was associated with signs of advanced disease, i.e., higher LV mass index, lower ejection fraction and greater LGE extent; 2) HyT2 was associated with a higher arrhythmic risk score, markers of arrhythmic burden (NSVT) and autonomic impairment (decreased heart rate variability), as shown by 24-h ECG recordings; and 3) HyT2 was the best predictor of NSVT among all CMR-derived and clinical parameters. These results suggest that the presence of myocardial edema, which was identified by HyT2 in HCM patients, is linked to disease progression and arrhythmogenesis.

HyT2 was detected in 95% of patients with NSVT during the 24-h ECG recording. Indeed, NSVT detection is considered a relevant arrhythmic risk marker in patients with HCM as well as ischemic and non-ischemic cardiomyopathies [27]–[29]. The presence of HyT2 was also associated with decreased heart rate variability, which suggests a sympathovagal imbalance, with decreased vagal tone, net sympathetic predominance, and subsequent cardiac electrical instability. Low heart rate variability is generally associated with an increased risk of sudden death in ischemic and non-ischemic heart disease [30]–[31] and the risk of arrhythmic events in patients with HCM [32]. In addition, the occurrence of NSVT is a risk factor for sudden cardiac death. However, conventional ambulatory ECG recordings, which last up to 24 h, may underestimate the arrhythmic risk of HCM patients or miss NSVT events due to their relatively short duration. Conversely, HyT2 may be detected >1 month after the acute event, and this timing provides a clinical advantage because HyT2 detection may permit the identification of patients with electrical instability, who have a higher arrhythmic risk and require strict clinical surveillance and enhanced therapeutic effort, even when NSVT is absent on ECG recordings.

Although HyT2 is a sign of myocardial edema on CMR, this relationship remains incompletely understood [14]. In ischemic heart disease, myocardial edema occurs secondary to prolonged acute ischemic events [33]. In the setting of acute myocardial infarction, edema highlights the presence of ischemic but viable myocardium surrounding necrotic areas, and the ratio between edema and the extent of necrosis is used to assess the myocardial salvage index [34]. In myocarditis, HyT2 is considered a sign of active inflammation and is usually located in the subepicardial layer or in the midwall [35]. HyT2 usually lasts one month after a myocardial infarction, whereas it may be detected 6 months after myocarditis [36].

In our HCM patients, HyT2 was located in the midwall of hypertrophic myocardial segments and was localized with or without LGE. Previously, Melacini et al. hypothesized that these T2 abnormalities in HCM could be attributed to ischemia caused by microvascular dysfunction, impaired diastolic relaxation, mismatch between capillary density, myocardial tissue interstitial fibrosis and/or myocardial bridging [17]. In this setting, prolonged ischemia involving the hypertrophic myocardial segments may cause small intramural, rather than subendocardial, myocardial damage, which presents on CMR as both HyT2 and LGE. HyT2 may be detectable in the acute/subacute phase but may subsequently disappear, while LGE may persist as a chronic scar [17]. However, Frustaci and colleagues found histopathological evidence of acute myocarditis in biopsies in a significant fraction of their HCM cohort, which was related to the patient’s clinical status [37]; therefore, HyT2 may indicate the presence of inflammatory myocardial damage.

The hypothesis that microvascular disease and ischemia result in HyT2 is strongly supported by the observation that the area of HyT2 closely matched the region of hypoperfusion based on the first-pass gadolinium CMR technique [18]. Moreover, a global decrease in myocardial blood flow was previously demonstrated in patients with HCM in CMR and positron emission tomography (PET) studies [15]. Specifically, the extent of LGE was inversely related to the global myocardial blood flow, suggesting a close relationship between ischemic events and chronic myocardial damage [38]. Furthermore, repetitive episodes of ischemia could explain the rapid progression of myocardial fibrosis in HCM, as recently demonstrated [11]. In the current study, we detected HyT2 in 42% of patients with HCM. In particular, patients with HyT2 demonstrated higher LV mass indexes, lower ejection fractions and a greater extent of LGE than those without HyT2. Moreover, patients with HyT2 had more arrhythmic risk factors than those without. Together, these findings suggest the presence of more advanced disease in patients with HyT2. A higher LV mass index has also been associated with microvascular disease [39], a lower blood supply/demand ratio and increased interstitial fibrosis. These findings suggest that, in advanced disease, ischemic events may be more severe and prolonged than in the early stage and may cause myocardial damage, ranging from a reversible injury, such as HyT2, to an irreversible cell loss that eventually produces myocardial fibrosis. Moreover, these factors may represent the substrate for electrical instability, which may be triggered by prolonged ischemic events. As hypothesized in Coumel’s triangle theory, myocardial disarray, fibrosis and hypertrophy serve as arrhythmogenic substrates that necessitate a trigger to induce arrhythmic events [40]; these events may finally be elicited by either ischemia or inflammation, which are detected by CMR as HyT2.

The presence of LGE in patients with HCM may be considered relevant in terms of risk stratification, as recent reports have demonstrated that after a clinical follow-up of 3 years, patients with LGE had a worse prognoses than those without [8], [9]. However, LGE is usually detected in most HCM patients during the first CMR evaluation, with a reported prevalence of 60–90% [6], [41]. Thus, the specificity of LGE as a prognostic marker in HCM should be discussed. Moreover, once cardiac fibrosis develops, its progression is relatively rapid. Indeed, we recently demonstrated that the progression of LGE extent was fast and related to clinical worsening [11]. Thus, features other than LGE extent should be investigated as predictors of sudden cardiac death [42]; for example, the presence of extensive (>15% of LV mass) and diffuse LGE is currently indicated as an emerging new arrhythmic risk factor [43]. However, previous studies showed that an extent of LGE >15% of LV mass was related to depressed systolic function [41], which may contribute to progression to end-stage disease [44]. Other CMR techniques, such as T1 mapping and measurements of the extracellular volume, are currently under evaluation as prognostic factors for HCM.

Some limitations of the current study should be mentioned. First, HyT2 may be detected in conditions other than ischemia and inflammation, and the results of the current study did not allow us to understand the nature and etiology of HyT2 in HCM. Additionally, the mechanism underlying the association between HyT2 and ventricular arrhythmias remains unclear. Second, we assumed that HyT2 in HCM is a sign of acute, transient myocardial damage, although this assumption was based only on the observation of HyT2 in other cardiac diseases. Thus, further studies with serial CMR examinations should be performed to confirm the reversibility of HyT2 and the prognostic role of HyT2 in larger HCM populations.

In conclusion, in patients with HCM, the presence of HyT2 upon CMR examination is associated with more advanced disease, ventricular arrhythmias and signs of electrical instability. HyT2 was detected in 42% of patients with HCM, and it was the best predictor of NSVT during a 24-h Holter ECG recording. HyT2 was associated with decreased heart rate variability and a greater number of arrhythmic risk factors.
